# Supplementary material for: Quantifying cooperative multisite binding in the hub protein LC8 through Bayesian inference
Source: PLoS Comput Biol. 2023 Apr 21;19(4):e1011059. doi: 10.1371/journal.pcbi.1011059 (PMC10155966; doi:10.1371/journal.pcbi.1011059)
Supplement: S1 Fig — (a) MCMC traces consisting of 50 chains for all model parameters for a synthetic isotherm (Fig 3B). Chains are thinned by a factor of 50 for visibility. (b) One and two-dimensional marginal distributions for all model parameters for a synthetic isotherm (Fig 3B), with contours in the two-dimensional plots set at 95 (yellow), 75 (orange), 50(purple) and 25%(black) confidence. Red lines and dots indicate true values for the synthetic isotherm. Correlations between some parameters are apparent, including between concentrations, as well as between each concentration and the thermodynamic parameters, especially ΔH. (PDF) [file pcbi.1011059.s001.pdf]

(a)

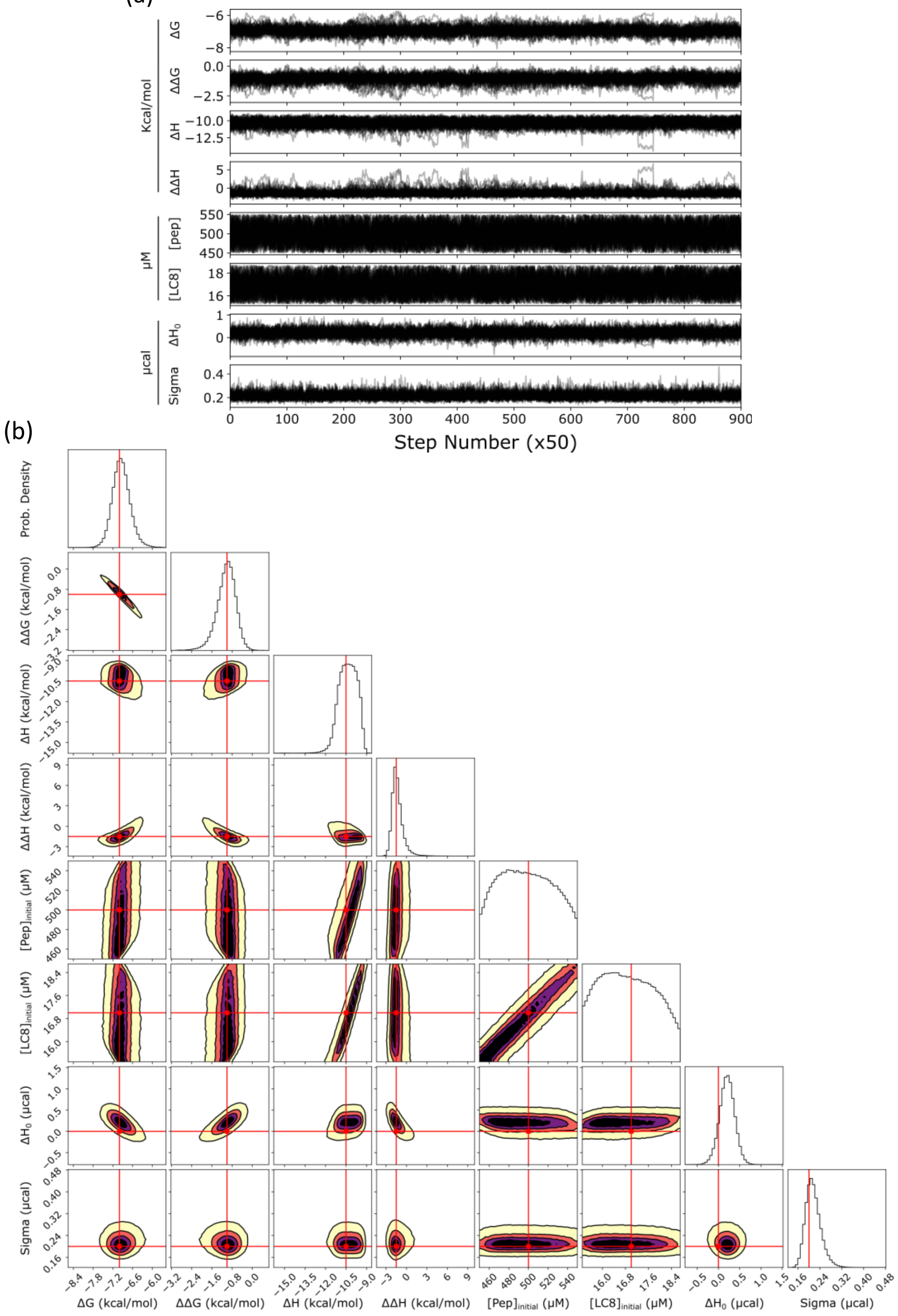

S1 Figure: MCMC traces and marginal distributions for all model parameters for a synthetic isotherm. (a) MCMC traces consisting of 50 chains for all model parameters for a synthetic isotherm (Fig. 3b). Chains are thinned by a factor of 50 for visibility. (b) One and two-dimensional marginal distributions for all model parameters for a synthetic isotherm (Fig. 3b), with contours in the two-dimensional plots set at 95 (yellow), 75 (orange), 50(purple) and 25%(black) confidence. Red lines and dots indicate true values for the synthetic isotherm. Correlations between some parameters are apparent, including between concentrations, as well as between each concentration and the thermodynamic parameters, especially ΔH.
