# Supplementary material for: Quantifying cooperative multisite binding in the hub protein LC8 through Bayesian inference
Source: PLoS Comput Biol. 2023 Apr 21;19(4):e1011059. doi: 10.1371/journal.pcbi.1011059 (PMC10155966; doi:10.1371/journal.pcbi.1011059)
Supplement: S2 Fig — Plots of predicted versus observed credibility for each thermodynamic parameter and analyte concentrations for our test synthetic isotherm conditions (Figs 3B and S1). Black circles are the complete model including concentrations, while red circles are from a simplified model with concentrations removed. Error bars are standard deviations across 1000 bootstrapped samples. (PDF) [file pcbi.1011059.s002.pdf]

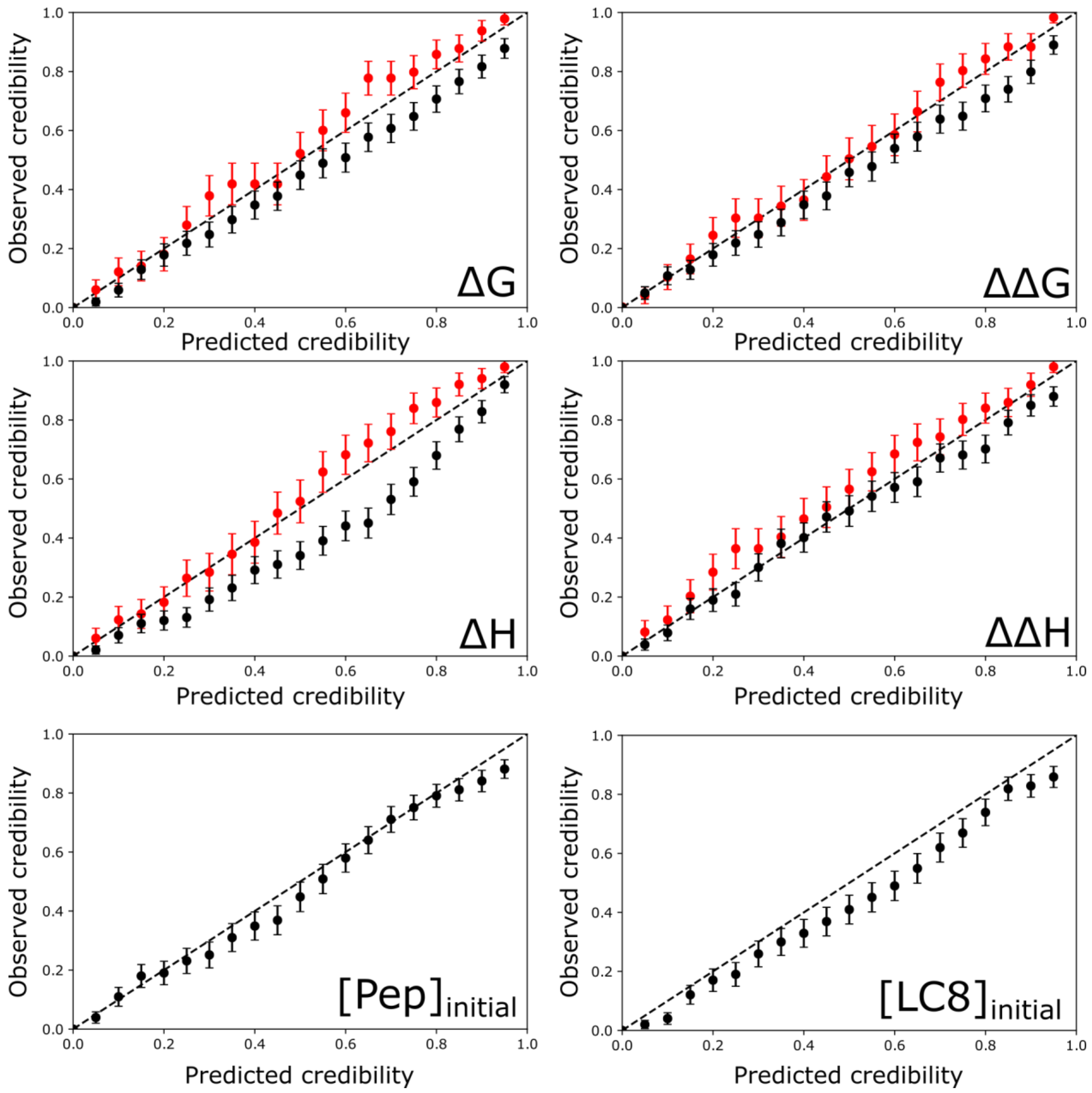

S2 Figure: Validation of uncertainty in credibility regions. Plots of predicted versus observed credibility for each thermodynamic parameter and analyte concentrations for our test synthetic isotherm conditions (Fig. 3b, S1 Fig.). Black circles are the complete model including concentrations, while red circles are from a simplified model with concentrations removed. Error bars are standard deviations across 1000 bootstrapped samples.
