# Supplementary material for: Quantifying cooperative multisite binding in the hub protein LC8 through Bayesian inference
Source: PLoS Comput Biol. 2023 Apr 21;19(4):e1011059. doi: 10.1371/journal.pcbi.1011059 (PMC10155966; doi:10.1371/journal.pcbi.1011059)
Supplement: S3 Fig — Each plot shows a set of either ΔG and ΔΔG or ΔH and ΔΔH, along with the ‘total’ value for that parameter, i.e. 2ΔG+ΔΔG or 2ΔH+ΔΔH. The distributions for this sum value are often narrower than the individual parameters, as the total enthalpy and free energy of binding can be determined with higher precision from a given isotherm than the individual values. ΔG,ΔH are the energy and enthalpy of binding step 1, while ΔG+ΔΔG,ΔH+ΔΔH are the energy and enthalpy of binding step 2, making the total values reported here the energy and enthalpy of both binding steps combined. (PDF) [file pcbi.1011059.s003.pdf]

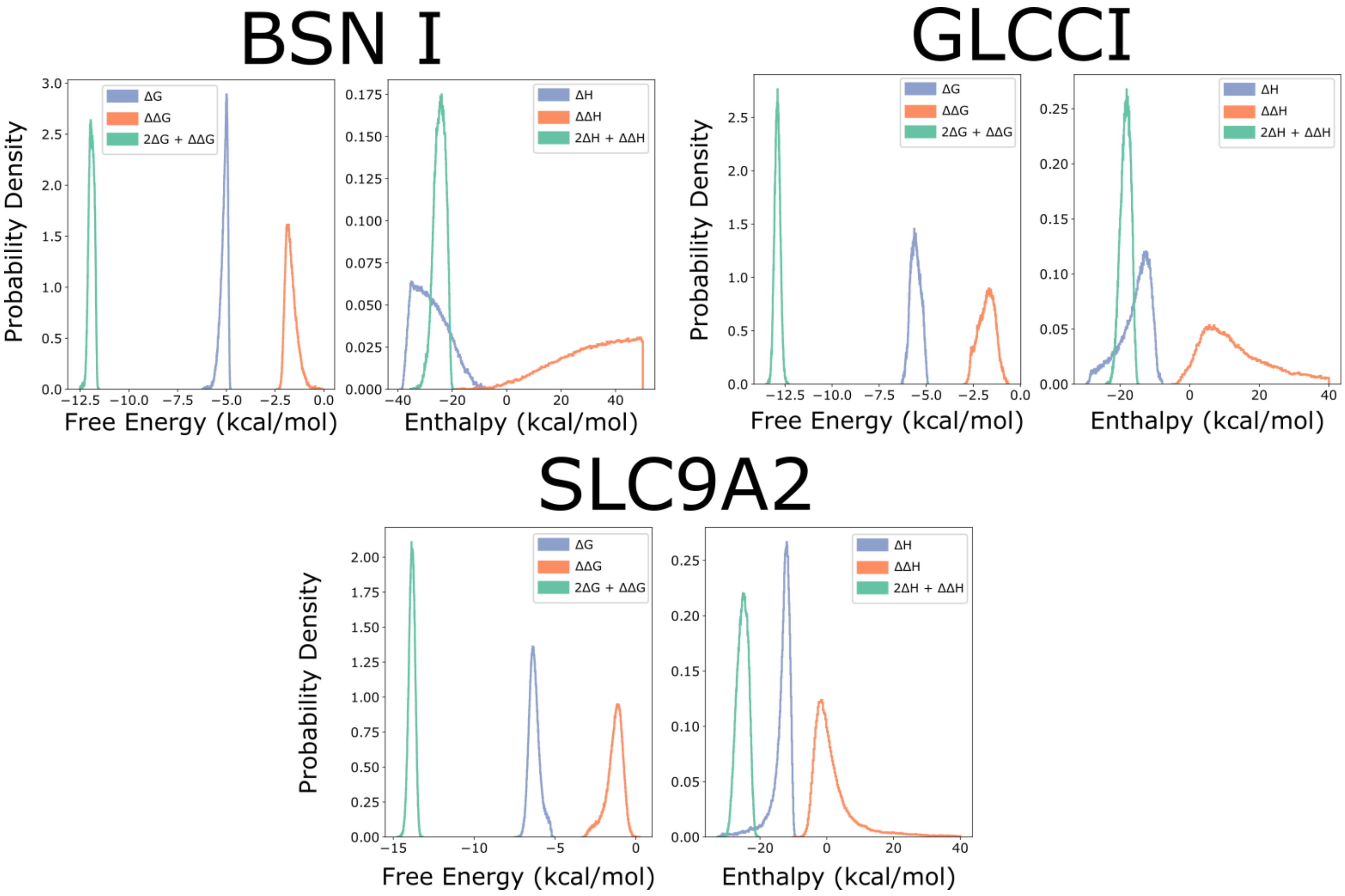

S3 Figure: Distributions of thermodynamic parameters plotted with total free energies and enthalpies. Each plot shows a set of either ΔG and $\Delta\Delta G$ or ΔH and $\Delta\Delta H$, along with the 'total' value for that parameter, i.e. $2\Delta G+\Delta\Delta G$ or $2\Delta H+\Delta\Delta H$. The distributions for this sum value are often narrower than the individual parameters, as the total enthalpy and free energy of binding can be determined with higher precision from a given isotherm than the individual values. ΔG,ΔH are the energy and enthalpy of binding step 1, while $\Delta G+\Delta\Delta G$,$\Delta H+\Delta\Delta H$ are the energy and enthalpy of binding step 2, making the total values reported here the energy and enthalpy of both binding steps combined.
