# Supplementary material for: Quantifying cooperative multisite binding in the hub protein LC8 through Bayesian inference
Source: PLoS Comput Biol. 2023 Apr 21;19(4):e1011059. doi: 10.1371/journal.pcbi.1011059 (PMC10155966; doi:10.1371/journal.pcbi.1011059)
Supplement: S4 Fig — Distributions are taken from models on an identical synthetic isotherm generated from parameters ΔG = -7, ΔΔG = -1, ΔH = -10, ΔΔH = -1.5, [peptide]initial = 500, [LC8]initial = 17, ΔH0 = 0 and sigma = 0.2. All priors are identical except for [peptide]initial, where the uniform prior model (red) was run with a ±10% of stated value uniform prior, and the normal prior model (black) was run with a normal distribution prior with standard deviation = 1% of stated value. (PDF) [file pcbi.1011059.s004.pdf]

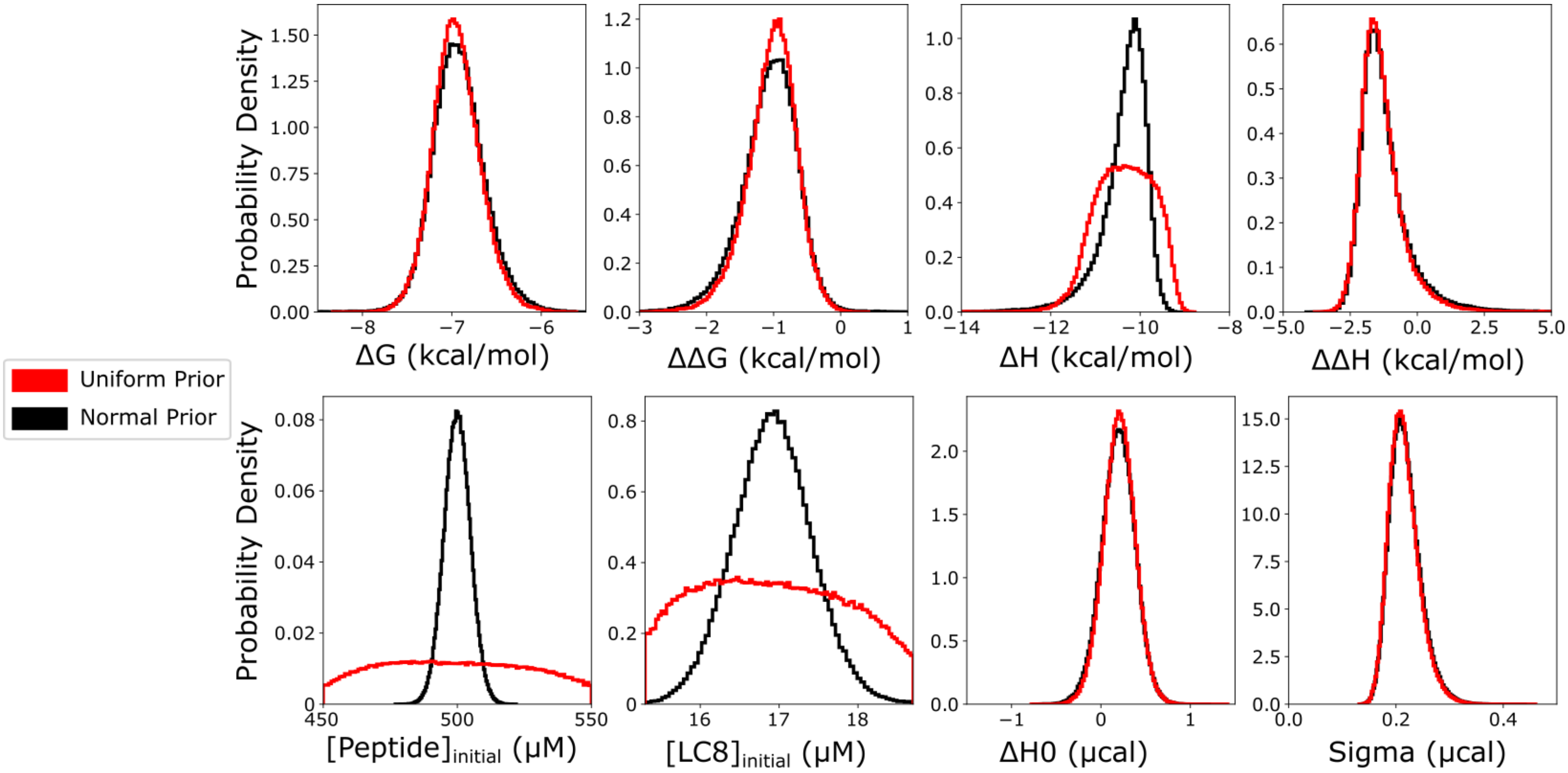

S4 Figure: Marginal distributions comparing models with uniform and normal-distribution priors. Distributions are taken from models on an identical synthetic isotherm generated from parameters ΔG = -7, $\Delta\Delta G$ = -1, ΔH = -10, $\Delta\Delta H$ = -1.5, $[\text{peptide}]_{initial}$ = 500, $[\text{LC8}]_{initial}$ = 17, ΔH_0 = 0 and sigma = 0.2. All priors are identical except for [peptide]initial, where the uniform prior model (red) was run with a ±10% of stated value uniform prior, and the normal prior model (black) was run with a normal distribution prior with standard deviation = 1% of stated value.
