# Supplementary material for: Quantifying cooperative multisite binding in the hub protein LC8 through Bayesian inference
Source: PLoS Comput Biol. 2023 Apr 21;19(4):e1011059. doi: 10.1371/journal.pcbi.1011059 (PMC10155966; doi:10.1371/journal.pcbi.1011059)
Supplement: S5 Fig — Distributions are taken from models on an identical synthetic isotherm generated from parameters ΔG = -8, ΔH = -12, [X]initial = 500, [M]initial = 34, ΔH0 = 0 and sigma = 0.2. Model priors ar0e uniform distributions of varied width in each plot for [X]initial and [M]initial, varied from ±1% to ±50%. (PDF) [file pcbi.1011059.s005.pdf]

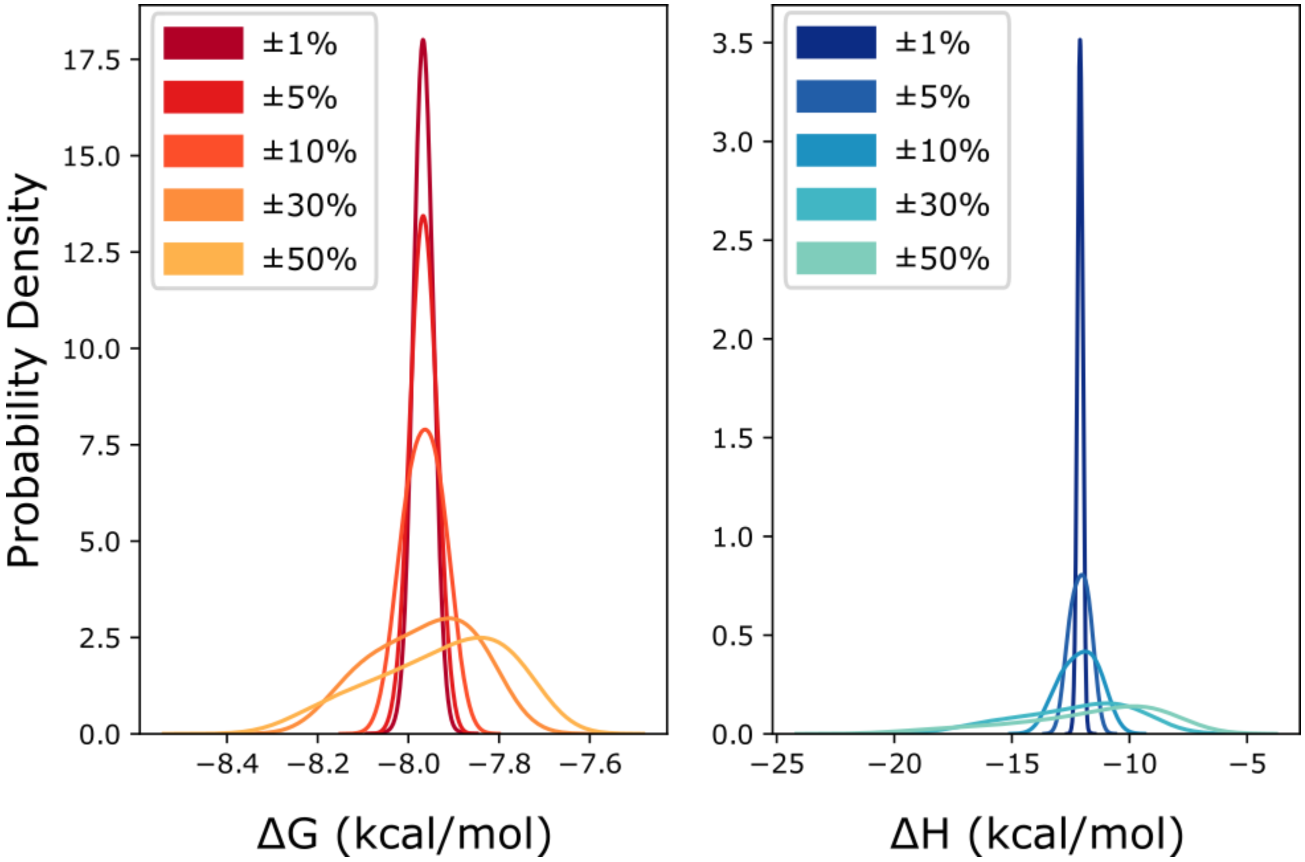

S5 Figure: Effect of concentration priors on marginal posterior distributions for thermodynamic parameters in a 1:1 binding model. Distributions are taken from models on an identical synthetic isotherm generated from parameters ΔG = -8, ΔH = -12, $[X]_{initial}$ = 500, $[M]_{initial}$ = 34, ΔH_0 = 0 and sigma = 0.2. Model priors ar0e uniform distributions of varied width in each plot for $[X]_{initial}$ and $[M]_{initial,}$ varied from ±1% to ±50%.
