# Supplementary material for: Quantifying cooperative multisite binding in the hub protein LC8 through Bayesian inference
Source: PLoS Comput Biol. 2023 Apr 21;19(4):e1011059. doi: 10.1371/journal.pcbi.1011059 (PMC10155966; doi:10.1371/journal.pcbi.1011059)
Supplement: S6 Fig — Each model replicate is run on an identical isotherm with a different random seed dictating random starts for MCMC chains and trial move selections. Each model returns near-identical marginal distributions. (PDF) [file pcbi.1011059.s006.pdf]

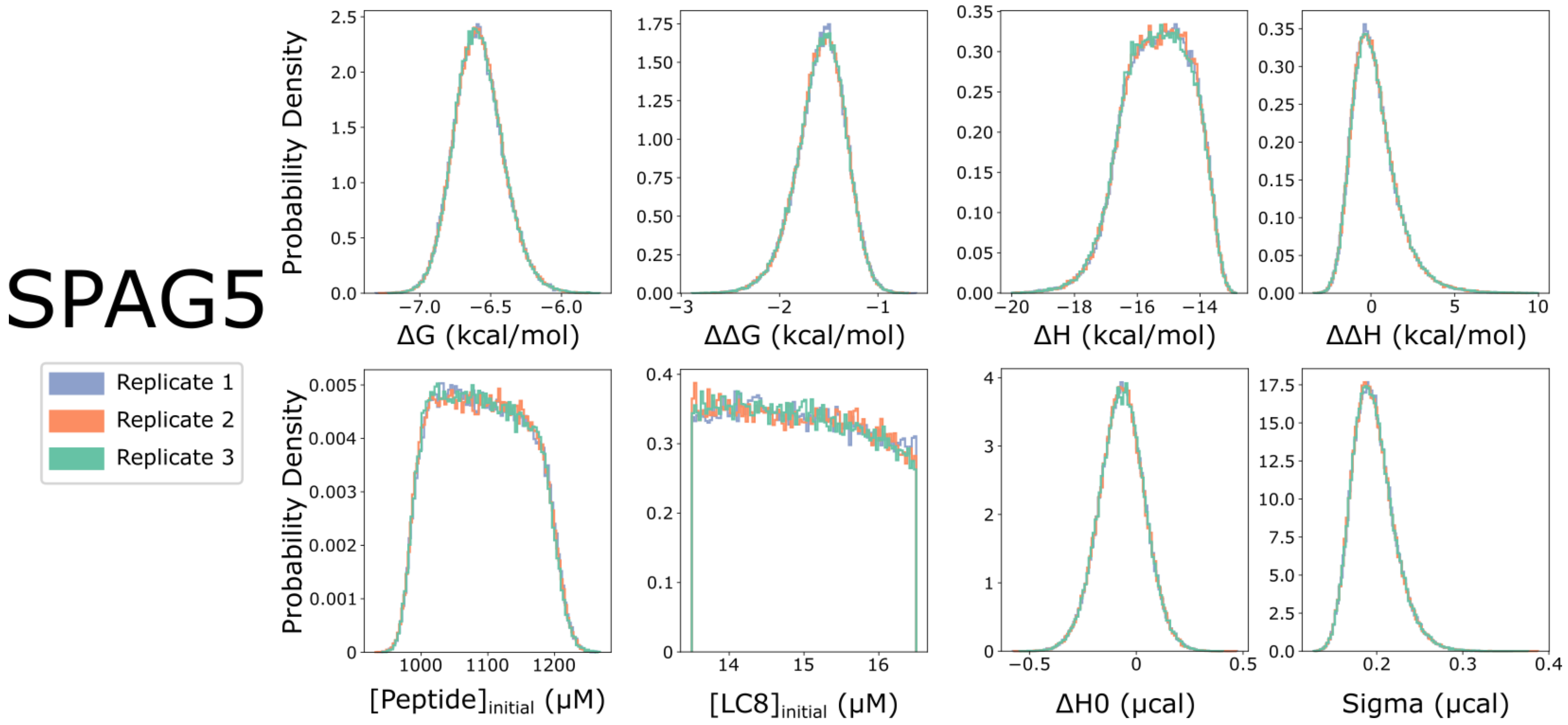

S6 Figure: Example marginal distributions of replicate models for the LC8-SPAG5 interaction. Each model replicate is run on an identical isotherm with a different random seed dictating random starts for MCMC chains and trial move selections. Each model returns near-identical marginal distributions.
