# Supplementary material for: Quantifying cooperative multisite binding in the hub protein LC8 through Bayesian inference
Source: PLoS Comput Biol. 2023 Apr 21;19(4):e1011059. doi: 10.1371/journal.pcbi.1011059 (PMC10155966; doi:10.1371/journal.pcbi.1011059)
Supplement: S7 Fig — Marginal distributions for BSN I, SLC9A2, and GLCCI are shown, each of which has wide 1D distributions for both ΔH and ΔΔH. Enthalpy parameters are closely correlated, resulting in a diagonal two-dimensional distribution within the enthalpy space. (PDF) [file pcbi.1011059.s007.pdf]

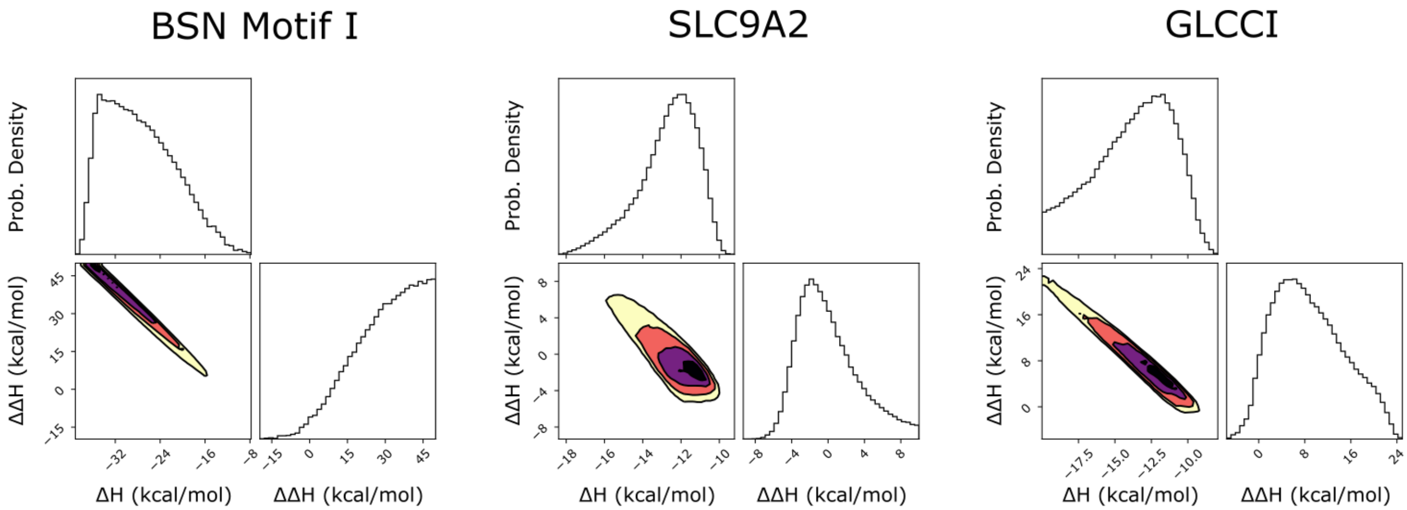

S7 Figure: two dimensional marginal distributions of enthalpy for selected isotherms. Marginal distributions for BSN I, SLC9A2, and GLCCI are shown, each of which has wide 1D distributions for both ΔH and ΔΔH. Enthalpy parameters are closely correlated, resulting in a diagonal two-dimensional distribution within the enthalpy space.
