# Supplementary material for: Quantifying cooperative multisite binding in the hub protein LC8 through Bayesian inference
Source: PLoS Comput Biol. 2023 Apr 21;19(4):e1011059. doi: 10.1371/journal.pcbi.1011059 (PMC10155966; doi:10.1371/journal.pcbi.1011059)
Supplement: S8 Fig — Distributions for each individual isotherm and distributions for the global model are shown in purple, orange and green respectively. While the global model improves precision in determined parameters in some cases (e.g. GLCCI), in others it appears to follow the shape of the distributions for individual isotherms (e.g. BSN I). (PDF) [file pcbi.1011059.s008.pdf]

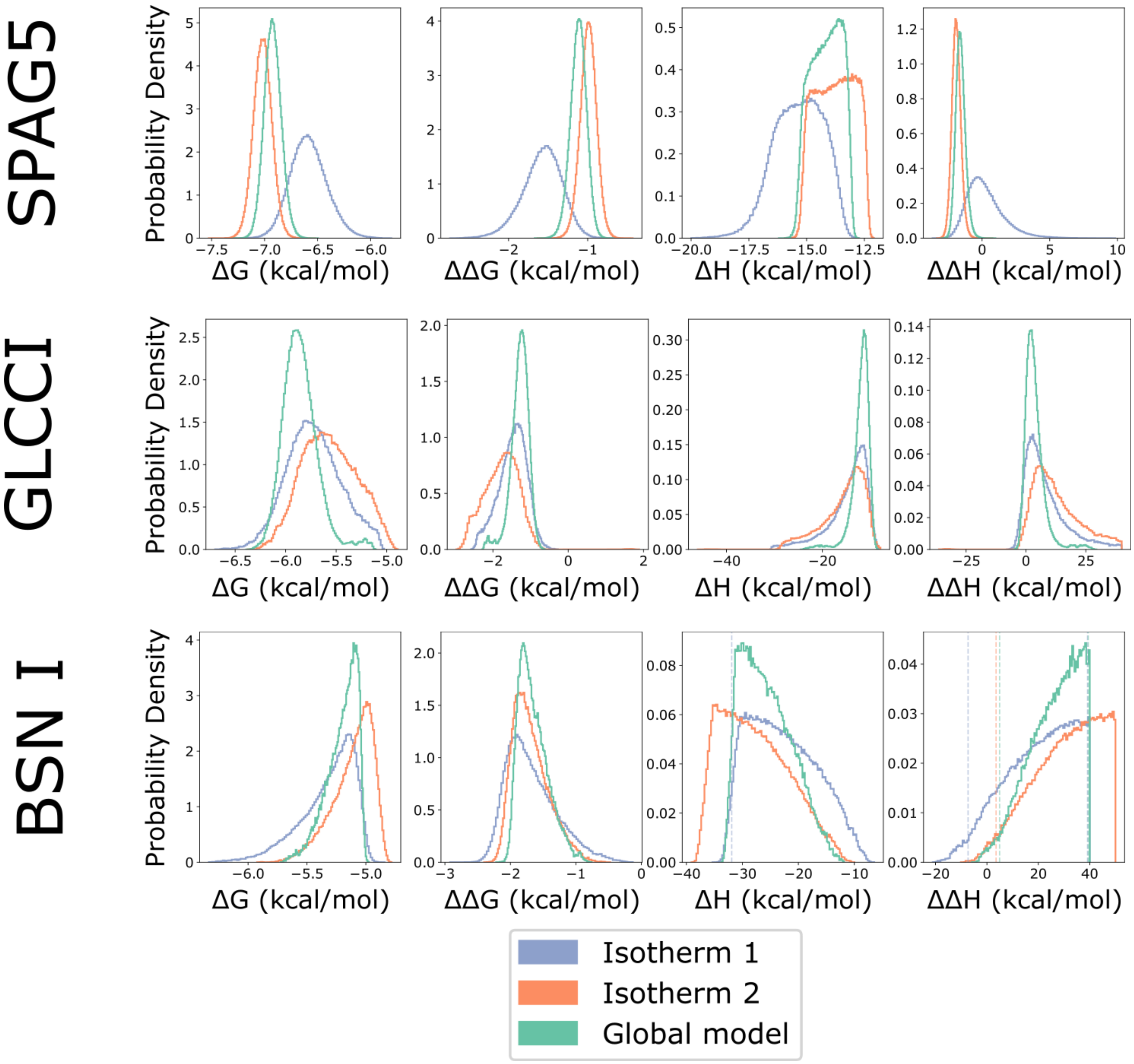

S8 Figure: Marginal distributions for thermodynamic parameters for individual and global models for three LC8-peptide interactions. Distributions for each individual isotherm and distributions for the global model are shown in purple, orange and green respectively. While the global model improves precision in determined parameters in some cases (e.g. GLCCI), in others it appears to follow the shape of the distributions for individual isotherms (e.g. BSN I).
