# Supplementary material for: Quantifying cooperative multisite binding in the hub protein LC8 through Bayesian inference
Source: PLoS Comput Biol. 2023 Apr 21;19(4):e1011059. doi: 10.1371/journal.pcbi.1011059 (PMC10155966; doi:10.1371/journal.pcbi.1011059)
Supplement: S9 Fig — Distributions for each individual isotherm and distributions for the global model are shown in green, orange, and purple respectively. (PDF) [file pcbi.1011059.s009.pdf]

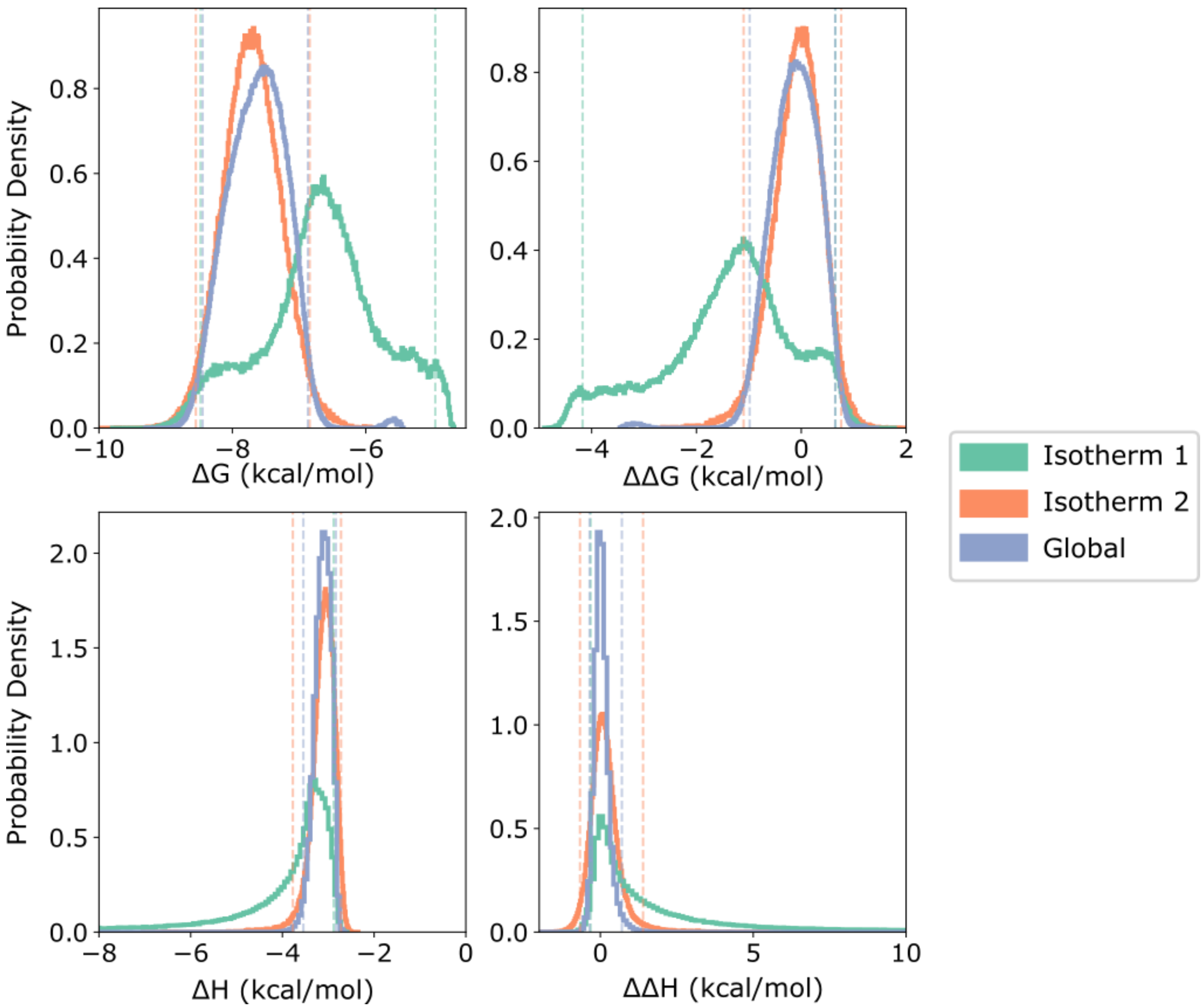

S9 Figure: Marginal distributions for thermodynamic parameters for IC-NudE binding isotherms. Distributions for each individual isotherm and distributions for the global model are shown in green, orange, and purple respectively.
