# Supplementary material for: Quantifying cooperative multisite binding in the hub protein LC8 through Bayesian inference
Source: PLoS Comput Biol. 2023 Apr 21;19(4):e1011059. doi: 10.1371/journal.pcbi.1011059 (PMC10155966; doi:10.1371/journal.pcbi.1011059)
Supplement: S10 Fig — (a) one-dimensional distributions for thermodynamic parameters for synthetic isotherms (ΔG = -5.1, ΔΔG = -1.7, ΔH = -11, ΔΔH = -2) generated with cell concentrations of 17 (orange) and 70 μM (blue and green). Syringe concentrations are 900 μM and 2000 μM respectively. The orange and blue isotherms are generated with noise taken from a gaussian distribution of width σ = 0.2 μcal, while the green is generated with σ = 0.8 ucal. (b) two-dimensional marginal distributions for the same models as (a), in the ΔH-ΔΔH dimension. Contours are drawn at 95 and 50% probability density. While raising the synthetic experimental concentration dramatically improves precision in all model parameters, much of this is due to the increased S/N ratio associated with the higher concentration. Scaling synthetic model noise with the increase in cell concentration reduces the precision of model enthalpies, although they are still narrower than the distributions for the low concentration isotherm. (PDF) [file pcbi.1011059.s010.pdf]

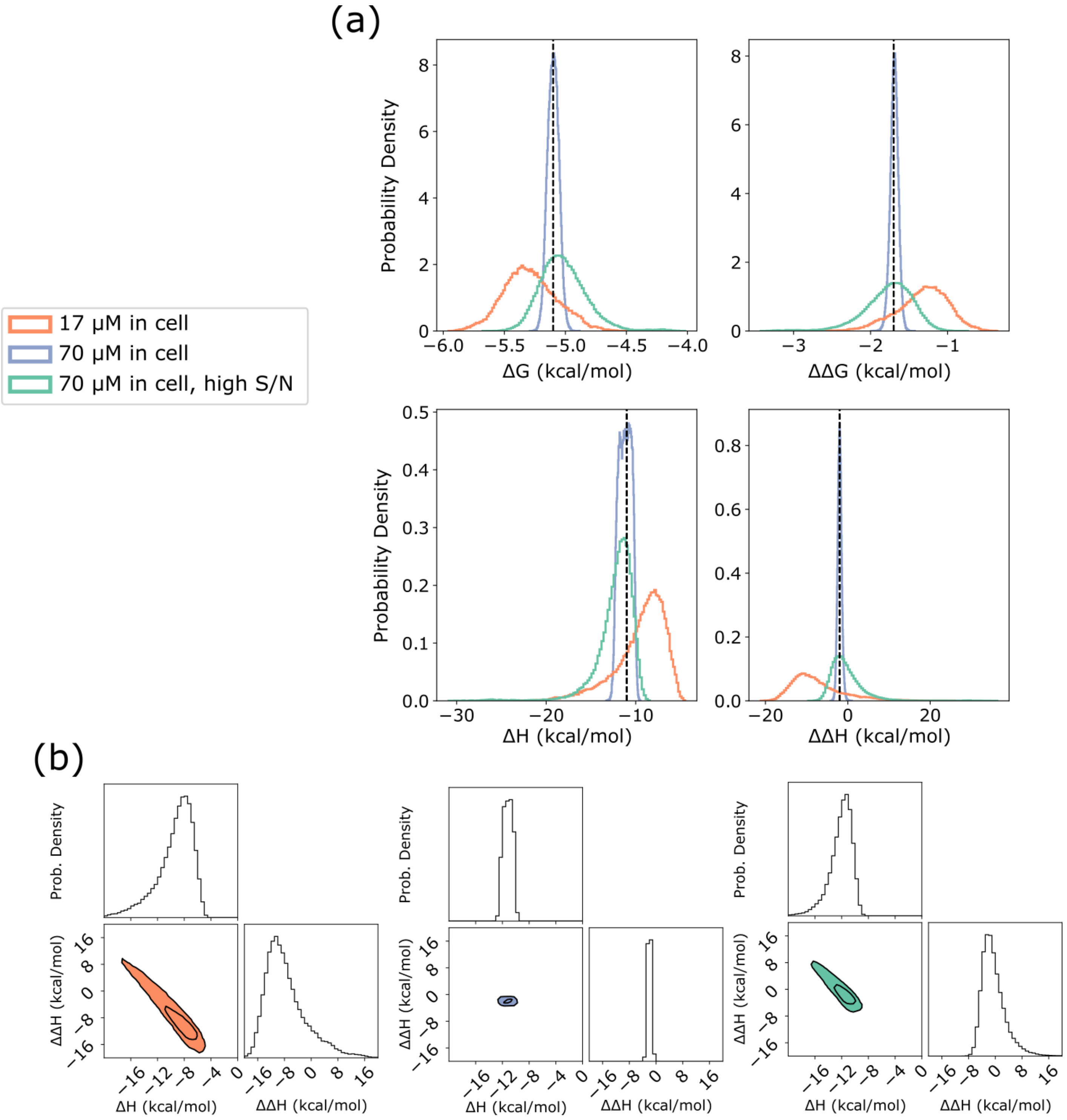


**S10 Figure: Marginal distributions for thermodynamic parameters for BSN-like synthetic isotherms.** (a) one-dimensional distributions for thermodynamic parameters for synthetic isotherms ($\Delta$G = -5.1, $\Delta\Delta$G = -1.7, $\Delta$H = -11, $\Delta\Delta$H = -2) generated with cell concentrations of 17 (orange) and 70 $\mu$M (blue and green). Syringe concentrations are 900 $\mu$M and 2000 $\mu$M respectively. The orange and blue isotherms are generated with noise taken from a gaussian distribution of width $\sigma$=0.2 $\mu$cal, while the green is generated with $\sigma$=0.8 ucal. (b) two-dimensional marginal distributions for the same models as (a), in the $\Delta$H-$\Delta\Delta$H dimension. Contours are drawn at 95 and 50% probability density. While raising the synthetic experimental concentration dramatically improves precision in all model parameters, much of this is due to the increased S/N ratio associated with the higher concentration. Scaling synthetic model noise with the increase in cell concentration reduces the precision of model enthalpies, although they are still narrower than the distributions for the low concentration isotherm.
