# Supplementary material for: Quantifying cooperative multisite binding in the hub protein LC8 through Bayesian inference
Source: PLoS Comput Biol. 2023 Apr 21;19(4):e1011059. doi: 10.1371/journal.pcbi.1011059 (PMC10155966; doi:10.1371/journal.pcbi.1011059)
Supplement: S1 Table — 95% credibility region from sampled posterior distributions for the ΔG sum(2ΔG+ΔΔG) and dH sum(2ΔH+ΔΔH) as well as the ratio of concentrations ([peptide]/[LC8]). Credibility regions for ΔG and ΔH sums are frequently narrower than the credibility regions for individual parameters (Table 1). (PDF) [file pcbi.1011059.s011.pdf]

Isotherm	∆G sum min	∆G sum max	∆H sum min	∆H sum max	conc ratio min	conc ratio max
SPAG5	-15.0	-14.5	-34	-28	71	75
BSN (I)	-12.2	-11.7	-29	-21	64	86
BSN (II)	-14.5	-14.1	-20	-17	61	66
SLC9A2	-14.2	-13.5	-29	-22	72	84
Ebola VP35	-15.7	-15.3	-27	-22	21	22
GLCCI	-13.2	-12.6	-21	-16	85	99
BIM	-18.2	-16.1	-26	-22	25	28

S1 Table: Credibility regions for 'sum' thermodynamic parameters and ratios of concentrations. 95% credibility region from sampled posterior distributions for the ∆G sum(2∆G+∆∆G) and dH sum(2∆H+∆∆H) as well as the ratio of concentrations ([peptide]/[LC8]). Credibility regions for ∆G and ∆H sums are frequently narrower than the credibility regions for individual parameters (Table 1).
