# Supplementary material for: Quantifying cooperative multisite binding in the hub protein LC8 through Bayesian inference
Source: PLoS Comput Biol. 2023 Apr 21;19(4):e1011059. doi: 10.1371/journal.pcbi.1011059 (PMC10155966; doi:10.1371/journal.pcbi.1011059)
Supplement: S2 Table — Table 1 in the main text contains equivalent information at ±10% LC8 concentration. Values delineate 95% Bayesian credibility regions from sampled posterior distributions, when modeled with ±20% priors for LC8 concentration. Distributions are largely very similar to those presented in Table 1, with a slight decrease in precision. BSN I, for which posterior distributions are significantly broader, is the only notable exception. (PDF) [file pcbi.1011059.s012.pdf]

Isotherm	ΔG min	ΔG max	ΔΔG min	ΔΔG max	ΔH min	ΔH max	ΔΔH min	ΔΔH max	-TΔS min	-TΔS max	-TΔΔS min	-TΔΔS max
SPAG5	-6.93	-6.2	-2.12	-1.13	-19.11	-12.59	-1.87	3.7	5.97	12.61	-5.78	0.69
BSN (I)	-7	-4.76	-2	0.28	-36.87	-7.57	-15.87	48.08	0.77	31.97	-50	16.02
BSN (II)	-7.09	-6.44	-1.22	-0.39	-6.41	-4.44	-9.75	-6.53	-2.22	-0.46	5.68	8.99
SLC9A2	-6.85	-5.47	-2.64	-0.53	-24.97	-9.75	-5.19	24.76	3.19	19.49	-27.42	4.6
Ebola VP35	-7.4	-6.81	-1.67	-0.92	-15.23	-10.29	-0.77	1.45	3.22	8.08	-3.11	-0.18
GLCCI	-6.11	-5.21	-2.27	-0.99	-19.49	-9.35	-0.52	21.35	3.43	14.12	-23.57	-0.48
BIM	-9.5	-6.95	-2.18	0.8	-13.5	-9.57	-3.17	-0.47	1.05	5.54	-0.93	2.32

S2 Table: Ranges for thermodynamic parameters for LC8-client binding when modeled with ±20% LC8 concentration. Table 1 in the main text contains equivalent information at ±10% LC8 concentration. Values delineate 95% Bayesian credibility regions from sampled posterior distributions, when modeled with ±20% priors for LC8 concentration. Distributions are largely very similar to those presented in Table 1, with a slight decrease in precision. BSN I, for which posterior distributions are significantly broader, is the only notable exception.
