# Supplementary material for: Quantifying cooperative multisite binding in the hub protein LC8 through Bayesian inference
Source: PLoS Comput Biol. 2023 Apr 21;19(4):e1011059. doi: 10.1371/journal.pcbi.1011059 (PMC10155966; doi:10.1371/journal.pcbi.1011059)
Supplement: S3 Table — (PDF) [file pcbi.1011059.s013.pdf]

Peptide	ΔG	ΔH	*n*
SPAG5	-8.0 ± 0.3	-15.10 ± 0.09	1.01 ± 0.004
BSN (I)	-7.3 ± 0.7	-12.8 ± 0.3	0.99 ± 0.02
BSN (II)	-7.9 ± 1.2	-9.8 ± 0.3	0.95 ± 0.02
SLC9A2	-7.7 ± 0.6	-11.52 ± 0.15	1.01 ± 0.01
Ebola VP35	-8.4 ± 0.4	-11.84 ± 0.06	1.00 ± 0.004
GLCCI	-7.3 ± 0.6	-10.8 ± 0.2	1.00 ± 0.015
BIM	-8.6 ± 0.7	-11.45 ± 0.09	1.02 ± 0.006

S3 Table: Binding parameters determined from identical sites model fits, as published in Jespersen et. al. (2019)(1).

References

1. Jespersen N, Estelle A, Waugh N, Davey NE, Blikstad C, Ammon YC, et al. Systematic identification of recognition motifs for the hub protein LC8. Life Sci Alliance. 2019 Aug;2(4):e201900366.
