# Supplementary material for: Quantifying cooperative multisite binding in the hub protein LC8 through Bayesian inference
Source: PLoS Comput Biol. 2023 Apr 21;19(4):e1011059. doi: 10.1371/journal.pcbi.1011059 (PMC10155966; doi:10.1371/journal.pcbi.1011059)
Supplement: S4 Table — (PDF) [file pcbi.1011059.s014.pdf]

Isotherm	# samples	# walkers	dG prior (kcal/mol)	dH prior (kcal/mol)	ddG prior (kcal/mol)	ddH Prior (kcal/mol)	X initial Prior (µcal)	M initial Prior (µcal)
Synthetic isotherm models	50,000	25-50	-3 to -10	-50 to 0	-4 to 4	-40 to 40	Varied - ±10% of stated unless otherwise noted	Varied - ±10% of stated unless otherwise noted
Single isotherm experimental models	100,000	50	-3 to -10	-50 to 0	-4 to 4	-40 to 40	± 10% of stated	Up to ±50% of stated
Two isotherm experimental models	200,000	50	-3 to -10	-50 to 0	-4 to 4	-40 to 40	± 10% of stated	Up to ±50% of stated

S4 Table: Model priors and sampling lengths for all isotherms.
