# Supplementary material for: Quantifying cooperative multisite binding in the hub protein LC8 through Bayesian inference
Source: PLoS Comput Biol. 2023 Apr 21;19(4):e1011059. doi: 10.1371/journal.pcbi.1011059 (PMC10155966; doi:10.1371/journal.pcbi.1011059)
Supplement: S1 Document — (PDF) [file pcbi.1011059.s015.pdf]

Quantifying cooperative multisite binding in the hub protein LC8 through Bayesian inference, Supplementary Information

Best practices for the application of Bayesian statistical models to isothermal titration calorimetry

Aidan B Estelle[1], August George[2], Elisar J Barbar*[1], Daniel M Zuckerman*[2]

[1]Department of Biochemistry and Biophysics, Oregon State University, Corvallis, Oregon 97331, United States

[2]Department of Biomedical Engineering, School of Medicine, Oregon Health and Science University, Portland, Oregon 97239, United states

This guide is intended to help anyone attempting to perform Bayesian inference on isothermal titration calorimetry (ITC) data for mono- or multivalent systems. The concentration degeneracy identified in the main paper makes the necessary Markov chain Monte Carlo (MCMC) even more challenging than usual. This guide is based on considerable effort experimenting with multiple algorithms and software packages.

Sampling

Adequate MCMC sampling is essential to the production of reliable parameter estimates from Bayesian inference. In our tests, we found the affine invariant sampling method (1) implemented in the python package EMCEE (2) performed by far the most effectively and efficiently for ITC modeling when including concentrations as model parameters. The affine method performs well in difficult parameter terrain (e.g. with a significant degeneracy), can run partially in parallel, and doesn't require the differentiation used in Hamilton Monte Carlo and related methods. The algorithm's employment of multiple MCMC walkers and construction of trial moves based on the locations of other walkers enables it to track narrow features in the landscape in an adaptive fashion.

EMCEE uses two 'hyperparameters' having to do with sampling: the number of walkers, and the number of steps each walker will take. While the EMCEE documentation recommends 3x the number of parameters as a minimum walker count, we found a higher number (e.g., 50 walkers for our 8 parameter model) improves the efficiency of sampling and reduces the likelihood of individual walkers "getting stuck," unable to move significantly relative to other walkers. For our model specifically, around 100,000 steps for each walker were sufficient for excellent sampling (confirmed by replicates), although this number is likely to be very model specific.

Although EMCEE's default trial move method, the 'stretch' move, performs robustly on ITC models, we found marginally increased performance through use of a mix at a ratio of 80:20% of the stretch move: the 'differential evolution snooker' move. The move performance may be model specific, however, so if possibly it is best to try a few trial move selection methods

Since there is no reliable way to predict how much sampling will be required, the quality of sampled posterior distributions must be checked. Methods for determining what qualifies as 'adequate' vary widely, such as measurement of r-hat (3), autocorrelation time (4) or effective sample size (5), but we suggest the use of replicate MCMC runs (each with its own seed) as a

metric for sufficient sampling. When sampling is adequate, the individual replicate posterior distributions for each parameter – or any observable of interest – should converge to be near-identical to each other (see Fig S6 of this manuscript for an example).

The number of sample steps required also scales roughly with the number of model parameters, as each parameter geometrically grows the size of the parameter space that must be explored. This means that any increase in model complexity, or application of global models with additional nuisance parameters will increase the number of required sampling steps – both the number of walkers and steps per walker, in EMCEE.

Global Models for multiple isotherms

When applying a 'global' model to multiple isotherms (with the aim of inferring a single model governing all isotherms), the most significant consideration is the addition of new nuisance parameters. The heat of dilution (ΔH_0 in our terminology), the model noise (A Gaussian distribution with mean = 0 and unknown variance sigma), and analyte concentrations are all isotherm specific parameters, so each isotherm will have its own set of these. In some cases, it may be possible to eliminate some parameters: e.g., If all isotherms are collected from the same stocks of protein, it could be acceptable to apply a 'global' prior for each concentration, rather than model each individual concentration for each isotherm. In general, though, a more complete model is better, and there is reduced risk of overfitting in a Bayesian framework relative to frequentist methodologies (6).

Experimentally, global models are best applied to isotherms with varied experimental conditions – particularly concentrations. While global models of technical-replicate isotherms are not useless, their ability to improve precision is limited, while isotherms containing non-overlapping information through variation of initial concentrations are more likely to improve global models (7, 8).

Concentration Priors

As discussed at length in the manuscript, individual concentrations cannot be determined through fitting or modeling. This means that limits set on the concentration ranges (i.e., by priors) are of particular significance to accurate modeling. Fortunately, the *ratio* of concentrations can be determined from the model (see main manuscript and Figs. 2 and 3), which functionally means that knowing only one concentration is adequate for a given experiment. Taking this into account, we advise that whichever experimental concentration can be determined with greater precision be used to limit the range of degenerate model solutions. The most naïve method to do this is to give the known concentration a uniform prior of ±X% around a measured value, where X is some measured uncertainty. Once that is set, the other analyte concentration can be set to as wide a prior as is needed: provided the two-dimensional posterior distribution shows a clear diagonal line within the parameter space, the model is able to find and sample along the degenerate line. If confidence in concentrations is particularly high, it may be reasonable to use gaussian distributions for priors, as it would likely be reasonable to expect Gaussian noise in concentration measurement.

Experimental considerations

While the normal considerations in setting up an ITC experiment apply, two additional considerations may apply for Bayesian modeling. First, as we discuss in the manuscript, when

modeling complex systems, there is no longer a single affinity around which to design experiments with regards to the ITC parameter c ($c = n[\text{cell}]/K_d$). In such cases, we believe global isotherms collected at multiple concentrations are likely to contain all the information necessary to extract all thermodynamic parameters, although we did not systematically explore this design consideration.

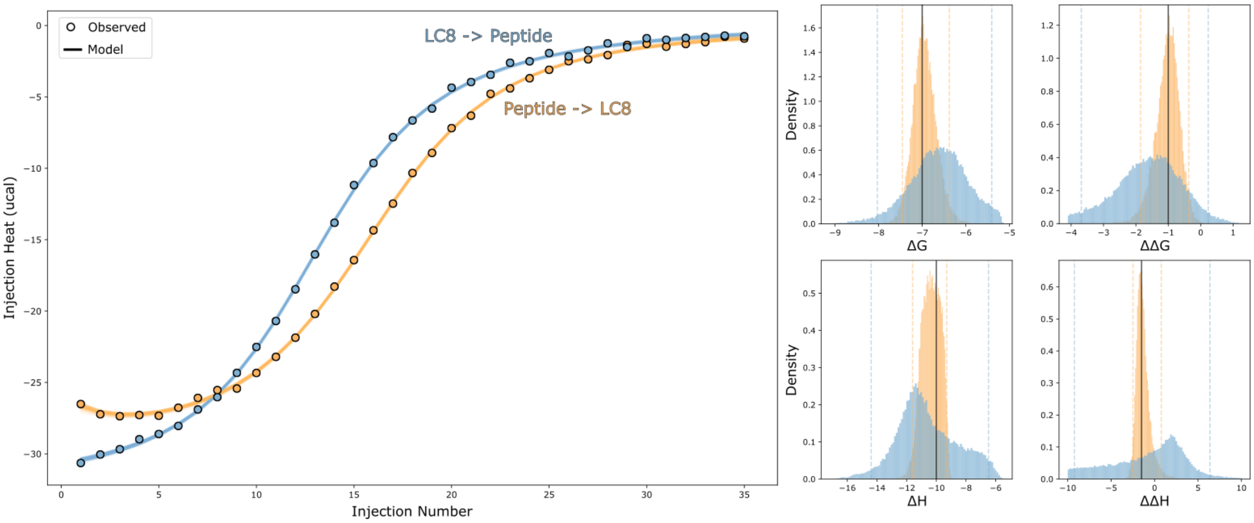

Figure 1: direction of injection impacts parameter determinability. Simulated isotherms of injection of LC8 into peptide (blue), and peptide into LC8 (yellow). Posterior distributions for injection of peptide into LC8 are significantly narrower than those for the LC8 titration.

Second, the information content of isotherms is dependent on the direction of injection. In tests for our specific 2-site system, we found from synthetic data that injecting the ligand into a solution of the protein with two sites returns significantly narrower distributions (on the order of 2-3 kcal/mol) than the reverse. We believe this is likely to depend on the binding model being tested and is best tested with synthetic isotherms prior to experimentation. Our example notebook, available at https://github.com/ZuckermanLab/Bayesian_ITC includes code for generating and modeling synthetic isotherms.

References

1. J. Goodman, J. Weare, Ensemble samplers with affine invariance. *CAMCoS* **5**, 65–80 (2010).

2. D. Foreman-Mackey, D. W. Hogg, D. Lang, J. Goodman, emcee : The MCMC Hammer. *Publications of the Astronomical Society of the Pacific* **125**, 306–312 (2013).

3. A. Gelman, D. B. Rubin, Inference from Iterative Simulation Using Multiple Sequences. *Statistical Science* **7**, 457–472 (1992).

4. M. Wallerberger, Efficient estimation of autocorrelation spectra (2019) (June 10, 2022).

5. V. Elvira, L. Martino, C. P. Robert, Rethinking the Effective Sample Size. *Int Statistical Rev*, insr.12500 (2022).

6. A. Gelman, *et al.*, Bayesian Data Analysis Third edition. 677.

7. M. V. C. Cardoso, *et al.*, CALX-CBD1 Ca2+-Binding Cooperativity Studied by NMR Spectroscopy and ITC with Bayesian Statistics. *Biophysical Journal* **119**, 337–348 (2020).

8. H. Duvvuri, L. C. Wheeler, M. J. Harms, pytc: Open-Source Python Software for Global Analyses of Isothermal Titration Calorimetry Data. *Biochemistry* **57**, 2578–2583 (2018).
